# An analysis of the prevalence and risk factors of deep vein thrombosis in non-fracture patients awaiting total hip arthroplasty: a retrospective study of 1244 cases

**DOI:** 10.1186/s13018-023-04488-9

**Published:** 2024-01-22

**Authors:** Yao Yao, Senlin Chai, Liang Qiao, Qing Jiang, Rong Xu

**Affiliations:** 1 State Key Laboratory of Pharmaceutical Biotechnology, Division of Sports Medicine and Adult Reconstructive Surgery, Department of Orthopedic Surgery, Nanjing Drum Tower Hospital, The Affiliated Hospital of Nanjing University Medical School, 321 Zhongshan Road, Nanjing, 210008 Jiangsu People’s Republic of China; 2 Branch of National Clinical Research Center for Orthopedics, Sports Medicine and Rehabilitation, Nanjing, People’s Republic of China; 3 Department of Orthopedics, The Second Affiliated Hospital of XuZhou Medical University, Xuzhou, Jiangsu People’s Republic of China; 4 Department of Rehabilitation Medicine, Nanjing Drum Tower Hospital, The Affiliated Hospital of Nanjing University Medical School, 321 Zhongshan Road, Nanjing, 210008 Jiangsu People’s Republic of China

**Keywords:** Deep vein thrombosis, D-dimer, Arthroplasty, Hip, Ultrasound

## Abstract

**Background:**

Deep vein thrombosis (DVT) has been one of the most dangerous complications in total hip arthroplasty (THA). If a patient’s pre-surgical DVT is overlooked, it can easily be mistaken for a post-operative thrombus and lead to an increased risk of DVT during and after surgery. This retrospective study was to explore the incidence and associated risk factors of deep vein thrombosis (DVT) in non-fracture patients before total hip arthroplasty (THA).

**Methods:**

From September 2015 to September 2020, 1242 patients admitted for THA were enrolled with 1120 patients (90.2%) for primary THA and 122 patients (9.8%) for revision THA. An experienced sonographer performed a bedside ultrasound to detect DVT in bilateral lower limbs preoperatively. Univariate and logistic regression analysis was performed to identify the independent risk factors.

**Results:**

38 patients (3.1%) were detected with preoperative DVT. Univariate analysis showed that age (*P* < 0.001), D-dimer level (*P* = 0.002), female patients (*P* = 0.016), revision THA (*P* < 0.001), Barthel Index score (*P* = 0.010) were significantly associated with preoperative DVT. In subgroup comparison, the incidence of DVT increased with age significantly (*P* < 0.001) and D-dimer level (*P* < 0.001). In logistic regression analysis, age ≥ 75 years old (odds ratio [OR] 3.678, 95% CI [2.197–18.721], *P* < 0.001), gender (OR 2.709, 95% CI [1.244–5.896], *P* = 0.012), higher D-dimer ≥ 0.5 mg/l (OR 6.841, 95% CI [2.197–18.721], *P* < 0.001) and revision THA (OR 2.240, 95% CI [1.143–5.372], *P* = 0.05) were confirmed as the independent risk factors.

**Conclusions:**

The incidence of preoperative DVT in non-fracture patients was 3.1%, with 2.4% in primary THA and 9.0% in revision THA. Age ≥ 75 years old, female, D-dimer ≥ 0.5 mg/l, and revision THA were independent risk factors. When evaluating the risk factors associated with thrombus formation preoperatively, it is important to take these into account before surgery.

## Introduction

Total hip arthroplasty (THA) is a surgical treatment that has been often used for end-stage hip disorders and can significantly reduce pain, restore function, and improve quality of life [1]. Over the past twenty years in China, there has been a dramatic increase in the number of patients undergoing THA. According to the Annual Data Report on Artificial Joints in China, it was estimated that the number of THA cases increased from the number 168,040 to 577,153 between 2011 and 2019 [2]. Despite its benefits, the procedure can potentially result in major postoperative complications such as venous thromboembolism (VTE), periprosthetic joint infection, fracture, dislocation, and implant failure [3–6].

VTE including deep vein thrombosis (DVT) and pulmonary embolism (PE), has been one of the most dangerous complications in major orthopedic surgery. It is estimated that the incidence of DVT and symptomatic PE following THA can range from 42 to 57% and from 2 to 5% respectively without thromboprophylaxis [7, 8]. The guidelines have recommended screening for DVT, using mechanical devices perioperatively, early mobilization as soon as possible after surgery, and using anticoagulants prophylactically for DVT prevention [9, 10]. In recent years, with the popularization of imaging examinations, more scholars have paid attention to the occurrence of DVT before joint replacement. For hip arthroplasty, most of the related research has focused on the cases of patients with femoral neck fractures and a high incidence of preoperative DVT has been reported in these patients. Some non-fractured THA patients also had moderate or severe pain, weak muscles, and low activity levels. Moreover, several DVT-related factors [11–15], including obesity, advanced age, and associated comorbidities, were present before admission, suggesting that DVT may have been located in the deep veins before THA. However, there has been very little research focusing on the topic of preoperative DVT in non-fractured THA patients [16–18]. Furthermore, the epidemiological, pathogenesis characteristics and prevention strategies of VTE before THA have not been well understood.

Thus, to avoid complications that can result in serious mortality, it is imperative to investigate the contributing factors as well as their respective roles in the occurrence of preoperative DVT in non-fractured THA patients. The goals of this study were to: investigate the incidence of DVT in non-fractured patients scheduled for THA and evaluate the risk factors for DVT prior to THA (general information, medical history, laboratory parameters).

## Materials and methods

The study protocol was reviewed and approved by the Ethics Committee of the Drum Tower Hospital (Ethics Number: 2012029) and was conducted in accordance with the 1964 Helsinki Declaration. Informed consent was obtained from all study patients. The study started with clearance from the institutional review board. Patients who were admitted for THA were enrolled in this study. All cases were strictly screened by senior doctors and had indications for surgery. The criteria for exclusion were: (1) patients with hip fractures, hemopathy, bone tumors or coagulopathy, known VTE before admission; (2) B-ultrasound was not done before surgery; (3) Incomplete information for any other reason. From September 2015 to September 2020, 2076 patients awaiting hip arthroplasty were recorded in the clinical data, 836 patients were excluded for the reasons mentioned above. A standardized data collection form was used to collect clinical information from electronic medical records (by Yao Y). The following parameters were enrolled including general information, medical history, preoperative coagulation, and lipid metabolism parameters.

### Preoperative diagnosis and management of VTE

An experienced sonographer (Yao Y) performed a SonoSite bedside ultrasound (Sonosite, M-Turbo, USA) to detect DVT in bilateral lower limbs preoperatively. Both proximal veins (common femoral vein [CFV], superficial femoral vein [SFV] and popliteal vein [PV]) and distal veins (peroneal vein [PEV], posterior tibial vein [PTV], anterior tibial vein [ATV], and muscular vein [MV]) were evaluated by a standard operating procedure [19]. The following criteria were used to diagnose DVT: (1) thrombotic echoes in veins and failure of the venous segment to fully compress; (2) lack of flow or irregular flow on color Doppler and spectral analysis [19]. Positive signs of the Homans and Neuhof test were defined with symptomatic DVT. Spiral computed tomography pulmonary angiography was performed on patients exhibiting clinical findings such as chest pain, dyspnea, or oxygen deprivation.

For patients without DVT before surgery, routine physical prophylaxis was encouraged by nurses or physical therapists and chemoprophylaxis was not routinely used. If patients were diagnosed with fresh DVTs in the following axial veins (FV, PV, PEV, PTV, ATV), surgery would be postponed and an additional anticoagulant was used. The dosage was used with low Molecular Heparin 0.4 ml twice daily or rivaroxaban 10 mg twice daily.

For patients with serious perioperative events such as proximal thrombosis multidisciplinary consultation was negotiated to formulate treatment plans such as thrombolysis and inferior vena filter placement.

After surgery, comprehensive preventive measures including pharmacological anticoagulants, graduated elastic stockings and intermittent pneumatic compression were used according to the guidelines. Chemoprophylaxis included low molecular Heparin or direct oral anticoagulants. Intermittent pneumatic compression was used for 8 h per day. In general, pneumatic pumps were not used postoperatively in people with preoperative DVT to prevent more serious complications.

### Statistical analysis

The SPSS statistical package (SPSS 22.0, USA) was used to analyze all the data. Means ± standard deviation was calculated to describe continuous variables. According to the ultrasound, the diagnostic results were divided into the DVT group and the non-DVT group. Continuous variables (age, body mass index, prothrombin time, International Normalized Ratio, thrombin time, activated partial thromboplastin time, fibrinogen, preoperative D-dimer, Triglyceride, total cholesterol, high density lipoprotein cholesterol, low density lipoprotein cholesterol, apolipoprotein A, apolipoprotein B, Barthel index score) were compared by using *t-test*. Categorical variables (gender, diabetes mellitus, hypertension, history of malignancy, history of previous DVT, stroke, smoking history, surgery type) were compared by using the χ2 test. Moreover, a logistic regression analysis was carried out to determine the independent risk factors. It was considered statistically significant if the *P* value ≤ 0.05.

## Results

In total, there were 1242 patients enrolled with 512 males (42.8%) and 710 females (57.2%). 1120 patients (90.2%) were admitted for primary THA and 122 patients (9.8%) for revision procedure. Among them, the mean age was 59.37 ± 13.31 years (range from 18 to 94 years old) and the average BMI was 24.36 ± 3.78 kg/m^2^ (range 13.67–51.42 kg/m^2^). There were 454 patients (36.6%) who had a history of hypertension, and 112 patients (9.0%) who had a history of diabetes mellitus. The mean Barthel index score of the study subjects was 85 ± 13 (range from 20 to 100). 233 patients (18.7%) had symptoms for less than 1 year, 827 patients (66.5%) had symptoms between 1 and 10 years, and 184 patients (14.8%) had symptoms for more than 10 years. The average D-dimer before THA was 0.94 ± 1.50 mg/l (range 0.06–25.8 mg/l). In primary THA, the surgical diagnosis was necrosis in 578 patients (51.6%), osteoarthritis in 530 patients (47.3%), rheumatoid arthritis in 9 patients (0.8%) and other in 3 patients (0.3%).

38 patients (3.1%) were finally identified with preoperative DVT including 27 cases (2.4%) in primary THA and 11 cases (9.0%) in revision THA. Among them, 7 DVT (18.4%) cases occurred in the bilateral leg and 31 cases (81.6%) in the unilateral leg. According to the anatomical distribution, there were 2 DVT cases (5.3%) in proximal veins and 36 cases (94.7%) in distal veins with 1 DVT case (2.6%) located in the FV, 1 DVT case (2.6%) in the PV, 35 DVTs (92.1%) in soleal vein (SV) and 1 case (2.6%) in the PEV and SV (Table 1). Among them, surgery in one patient with popliteal vein thrombosis was postponed. After therapeutic treatment with rivaroxaban 10 mg twice daily, the patient went back to the hospital and underwent revision THA. Another proximal DVT was regarded as chronic DVT and surgery was performed without delay. One patient with received inferior vena filter placement preoperatively and then underwent THA surgery. All 35 patients with soleal vein thrombosis undergone THA without preoperative anticoagulant therapy. After surgery, comprehensive preventive measures including pharmacological anticoagulants, graduated elastic stockings and intermittent pneumatic compression were used according to the guidelines. No symptomatic PE and death occurred.

**Table 1:** Characteristics of deep venous thrombosis

Variables	No. of patients
Location (proximal DVT)	2
Bilateral	7
Anatomy	
FV	1
PV	1
PEV + SV	1
SV	35

DVT, deep vein thrombosis; FV, femoral vein; PV, Popliteal vein; PEV, Peroneal vein; SV, Soleal vein

Table 2 listed the result of the univariate analysis indicating that age (*P* < 0.001), age ≥ 75 years old (*P* < 0.001), D-Dimer level (*P* < 0.001), D-dimer ≥ 0.5 mg/l (*P* < 0.001), female patients (*P* = 0.016) and revision THA (*P* < 0.001) were identified as risk factors for preoperative DVT (*P* < 0.001). Barthel Index in the DVT group was significantly lower than that in the non-DVT group (*P* = 0.010). However, no real connection was discovered between other variables and preoperative DVT (*P* > 0.05). In addition, a subgroup analysis was conducted based on age (group 1 less than 60 years, group 2 between 60 and 75 years, group 3 equal to or greater than75 years) and D-dimer level (group 1 less than 0.5 mg/l, group 2 between 0.5 and 1 mg/l, group 3 equal to or greater than 1 mg/l), respectively. The incidence of DVT increased with age (0.7%, 3.3% and 11.3%) (*P* < 0.001) (Table 3) and D-dimer level (0.6%, 2.9%, and 8.3%) (*P* < 0.001) (Table 4).

**Table 2:** Comparison of characteristics of the study population

Variables	Total (N = 1242)	DVT group (N = 38)	Non-DVT group (N = 1204)	*P* value
Age (years)	59.37 ± 13.31	72.18 ± 8.73	58.97 ± 13.23	*P* < 0.001*
Age ≥ 75 years old	150 (12.1%)	17 (44.7%)	133 (11.0%)	*P* < 0.001*
Gender (female)	710 (57.2%)	29 (76.3%)	681 (56.6%)	*P* = 0.015*
BMI (kg/m^2^)	24.36 ± 3.78	23.92 ± 3.79	24.37 ± 3.78	*P* = 0.465
Hypertension	454 (36.6%)	18 (47.4%)	436 (36.2%)	*P* = 0.160
Diabetes mellitus	112 (9.0%)	3 (7.9%)	109 (9.1%)	*P* = 1.000
History of VTE	2 (0.2%)	1 (2.6%)	1 (0.1%)	*P* = 0.071
History of malignancy	34 (2.7%)	0	34 (2.8%)	*P* = 0.585
History of stroke	77 (6.2%)	4 (10.5%)	73 (6.1%)	*P* = 0.434
Smoking history	125 (10.1%)	0	125 (10.4%)	*P* = 0.069
Surgery type (revision THA)	122 (9.8%)	11 (28.9%)	111(9.2%)	*P* < 0.001*
PT(S)	11.37 ± 1.00	11.41 ± 0.83	11.37 ± 1.00	*P* = 0.781
INR	1.00 ± 0.11	1.00 ± 0.07	1.00 ± 0.11	*P* = 0.887
APTT(S)	27.76 ± 3.15	27.41 ± 3.33	27.77 ± 3.15	*P* = 0.489
TT(S)	18.27 ± 1.20	18.01 ± 1.22	18.28 ± 1.20	*P* = 0.180
Fbg(g/l)	3.11 ± 1.31	3.44 ± 2.47	3.09 ± 1.26	*P* = 0.389
D-dimer (mg/l)	0.94 ± 1.50	2.58 ± 3.12	0.88 ± 1.40	*P* = 0.002*
D-dimer ≥ 0.5 mg/l	608 (48.9%)	34 (89.5%)	574(47.6%)	*P* < 0.001*
TG (mmol/l)	1.43 ± 0.96	1.28 ± 0.75	1.43 ± 0.96	*P* = 0.335
TC (mmol/l)	4.36 ± 0.94	4.33 ± 0.89	4.36 ± 0.95	*P* = 0.836
HDL-C (mmol/l)	1.17 ± 0.34	1.25 ± 0.30	1.16 ± 0.34	*P* = 0.136
LDL-C (mmol/l)	2.55 ± 0.77	2.50 ± 0.68	2.55 ± 0.77	*P* = 0.685
apoA (mmol/l)	1.10 ± 0.27	1.15 ± 0.32	1.09 ± 0.27	*P* = 0.217
apoB (mmol/l)	0.82 ± 0.24	0.80 ± 0.20	0.82 ± 0.24	*P* = 0.481
Barthel index	85 ± 13	77 ± 19	85 ± 12	*P* = 0.010

DVT, deep vein thrombosis; BMI, body mass index; THA, total hip arthroplasty; PT, prothrombin time; INR, International Normalized Ratio; APTT, activated partial thromboplastin time; TT, thrombin time; Fbg, fibrinogen; TG, triglyceride; TC, total cholesterol; HDL, high density lipoprotein cholesterol; LDL-C, low density lipoprotein cholesterol; apoA, apolipoprotein A; apoB, apolipoprotein B

*P value ≤ 0.05

**Table 3:** Comparisons in age subgroups

Age (years)	< 60	≥ 60 < 75	≥ 75
DVT group	4	17	17
Non-DVT group	575	496	133
Incidence	0.7%	3.3%	11.3%
*P* value	< 0.001*		

DVT, deep vein thrombosis

*P value ≤ 0.05 was considered statistically significant

**Table 4:** Comparisons in D-dimer subgroups

D-dimer (mg/l)	< 0.5	≥ 0.5 < 1	≥ 1
DVT group	4	9	25
Non-DVT group	632	298	278
Incidence	0.6%	2.9%	8.3%
*P* value	< 0.001*		

DVT, deep vein thrombosis

*P value ≤ 0.05

Table 5 showed the result for logistic regression analysis, age ≥ 75 years old (odds ratio [OR] 3.678, 95% CI [2.197–18.721], *P* < 0.001), female (OR 2.709, 95% CI [1.244–5.896], *P* = 0.012), higher D-dimer ≥ 0.5 mg/l (OR 6.841, 95% CI [2.197–18.721], *P* < 0.001) and revision THA (OR 2.240, 95% CI [1.143–5.372], *P* = 0.05) were confirmed as the independent risk factors.

**Table 5:** Independent risk factors for preoperative DVT in logistic analysis

Variables	B	S.E.	Wald	*P* value	OR	95% CI
Age ≥ 75 years old	1.302	0.362	12.918	< 0.001*	3.678	2.197–18.721
Female	0.996	0.397	6.304	0.012*	2.709	1.244–5.896
D-dimer ≥ 0.5 mg/l	1.858	0.547	11.558	< 0.001*	6.841	2.197–18.721
Revision THA	0.806	0.411	3.842	0.050*	2.240	1.000–5.016
Barthel index score	− 0.010	0.011	0.833	0.361	0.990	0.968–1.012

DVT deep vein thrombosis; THA total hip arthroplasty; S.E., standard error; OR, odds ratio; CI, confidence interval

*P value ≤ 0.05

The risk factors for preoperative DVT in the revision THA group were analyzed in Table 6. The results showed that the average age in the DVT group was significantly older than that in non DVT group (74.55 ± 5.50 vs 64.31 ± 12.76 years old, *P* < 0.001). Female patients were more likely to have DVT than male patients in the revision THA group (*P* = 0.004). The average D-dimer value in the DVT group was higher than that in the non-DVT group, however, there was no significant difference between the two groups. Furthermore, in the subgroup analysis, the incidence of DVT in D-dimer ≥ 1 mg/l group was significantly higher than other two groups (*P* < 0.001). However, there was no statistical difference in the age subgroup analysis.

**Table 6:** Comparison of risk factors for preoperative DVT in revision THA group

Variables	DVT group	Non-DVT group	*P* value
	N = 11	N = 111	
Age (years)	74.55 ± 5.50	64.31 ± 12.76	*P* < 0.001*
Age (years)			*P* = 0.070
≥ 75	5 (45.5%)	31 (27.9%)	
60–75	6 (54.5%)	43 (38.7%)	
≤ 60	0	37 (33.3%)	
Gender (female)	10 (16.4%)	1 (1.6%)	*P* = 0.004*
D-dimer (mg/l)	3.74 ± 3.69	1.39 ± 1.57	*P* = 0.061
D-dimer (mg/l)			*P* < 0.001*
≥ 1	11 (100%)	51 (31.5%)	
0.5–1	0	25 (22.5%)	
< 0.5	0	25 (22.5%)	

*P value ≤ 0.05

## Discussion

There are limited studies on preoperative thrombosis before THA surgery in non-fracture patients, even though researchers have extensively researched the incidence, etiology, and prevention of VTE after THA. In this study, we concentrated on the formation of DVT and its risk factors in non-fracture patients prior to THA surgery. This finding indicated that a total of 38 patients (3.1%) had preoperative DVT before THA with 2.4% in primary THA and 9.0% in revision THA. Furthermore, as determined by this study, age ≥ 75 years old, female, D-dimer ≥ 0.5 mg/l, and revision THA were found to be risk factors for preoperative DVT.

In the literature, there have been three studies that concern the characteristics of preoperative DVT before THA. Imai et al. enrolled 224 patients who were admitted to the hospital for THA and found that 13 patients (5.8%) had preoperative DVTs [18]. Kawai et al. reviewed 500 patients for primary or revision THA and 26 DVTs (5.2%) were detected with 9 cases in proximal veins [17]. In a study of 505 patients, all of them underwent ultrasound scanning within a mean of 2 weeks before THA, and 62 (12.3%) preoperative DVTs were confirmed [16]. In contrast, the proportion of DVT in our study is a little lower than in previous studies. This may be explained by that patients included in the present study had a relatively younger age compared with Kawai et al. (59.33 vs 64.79 years), and lower preoperative D-dimer level compared with Imai et al.’s (0.94 vs 1.47 mg/l). Another reason could be attributed to different medical conditions. For example, according to the study of Wakabayashi et al., a total of 187 (37%) patients had a major surgery history, and the incidence of DVT in this subgroup reached 18.7%. The incidence was also lower than that in patients before knee arthroplasty. One study from Japan enrolled 322 patients admitted for total knee arthroplasty and 56 patients (17.4%) were screened to have preoperative DVT including 3 cases located in the proximal veins. In another study from Xiong et al., the authors analyzed 584 knee osteoarthritis patients awaiting undergoing total knee arthroplasty and found 40 patients (6.85%) had a preoperative DVT. This discrepancy may also be attributed to the younger age of patients undergoing THA in the present study, and another reason may be that muscle weakness in the calf was more noticeable in patients undergoing knee arthroplasty.

Numerous research articles have pointed out that old age has a dramatically higher risk of DVT in patients undergoing arthroplasty, based on a deep literature review [12, 20, 21]. One study included 1236 patients scheduled to undergo elective musculoskeletal surgery and the results showed that age 80 or older significantly increased the likelihood of preoperative DVT [22]. Another study performed on 521 end-stage knee osteoarthritis cases showed that the DVT group’s average age was nearly 4 years older than the non-DVT group’s average age (72.54 vs 68.65 years) [23]. According to the present study, a significant increase in preoperative DVT was also observed among older patients before THA, which was consistent with previous studies. Wakabayashi et al. [16] reported that the mean age in the preoperative DVT group was 5.8 years older than the non-DVT group before THA (67.5 vs 61.7 years, *P* < 0.001). Similarly, a retrospective study reported that age in preoperative DVT was 5.5 years older than the control group and age has a strong correlation with preoperative DVT in the multivariate logistic regression analysis (OR, 1.13; 95% CI 1.034–1.238; *P* < 0.007) [17]. Furthermore, the present study also showed the incidence of preoperative DVT was 0.7%, 3.3%, and 11.3% respectively in different subgroup analyses, and in the logistic analysis, the risk for preoperative in patients age 75 or older was 3.935 times higher than other younger patients. Therefore, before THA, clinicians should focus more on the risk of DVT in elderly patients.

In our studies, female gender was regarded as an independent risk factor for the development of preoperative DVT. Previous research has shown that female patients are more prone to suffer from VTE. In a study of the Korean population, it was observed that the incidence of postoperative DVT in women (r = 0.155, *P* = 0.021) was substantially higher than in men following THA [24]. In another study, the author identified 20 studies with a total of 7,892,585 patients undergoing THA or TKA. The result showed that the sex ratio (male/female) in the VTE group (0.492) was significantly different from that in the non-VTE group (0.623), which showed the risk of DVT is slightly higher in female patients than in male patients [25]. However, there is still a lack of understanding of the mechanisms contributing to the female sex having a high VTE amputation risk. Some possible reasons could explain this phenomenon. For one thing, the percentage of women who develop thrombosis following revision surgery is much higher than that of males (19.6% vs 2.1%), which may partly account for the difference. For another thing, it is possible that female patients were more prone to immobilization due to pain and muscle weakness.

According to the current study, a D-dimer level equal to or greater than 0.5 mg/l was significantly correlated with an increased risk of preoperative DVT. Furthermore, DVT incidence increased with the level of D-dimers (0.6%, 2.9%, and 8.3%, respectively) (*P* < 0.001). This result was similar to other findings [26, 27]. Although the D-dimer indicates greater sensitivity, it has a low level of specificity. In some studies, combining D-dimer and age can significantly improve the accuracy of DVT prediction. One study by Imai et al. showed that combining the D-dimer and age is a better method for the diagnosis of DVTs before THA, compared with using the D-dimer test alone [18]. In a study from Wu et al., 406 patients above 50 years old awaiting total joint arthroplasty were enrolled, and two D-dimer methods were compared to detect DVT effectiveness based on the standard ultrasound. Results showed that 39 patients had asymptomatic DVT and the age-adjusted D-dimer level had a higher accuracy in diagnosing DVT than D-dimer level alone. Furthermore, a significant improvement was also found in sensitivity and specificity when compared to the traditional method (76.8% vs 41%, 76.9% vs 65.8%) [28].

Patients undergoing revision surgery experience more postoperative problems than patients undergoing first surgery. However, few reports have been concerned with the development of DVT in revision arthroplasty surgery preoperatively. The present study indicated that the incidence of DVT before revision THA was higher than that in primary THA both in univariate and logistic analysis for the first time. In a previous report, Wakabayashi and colleagues found patients admitted for revision THA had an incidence of 19.3% of preoperative DVT and this result was significantly higher than primary THA group, However, in multiple linear regression analysis, revision THA was not found to be associated with preoperative DVT [16]. Another large number study analyzed the incidence of postoperative DVT including 7566 revision THA and 66,839 primary THA, and the authors found that the incidence after revision THA was 0.6%, which was higher than that after primary THA (0.4%) (*P* = 0.016). However, when confounding variables, revision surgery alone does not constitute an independent risk factor of DVT [29]. The reason for this result in our study may contribute to the older age, comorbidities, and preoperative poor mobility in patients awaiting primary THA.

The aforementioned study has an advantage over the prior literature in that it provides the first data on the prevalence of pre-existing DVT in non-fracture patients in China and contains the greatest number of patient cases undergoing THA, making it easier for doctors to detect and treat pre-existing DVT before surgery. Our study found preoperative lower limb thrombosis in 3.1% of patients preparing for THA. Before this, these patients were likely to be considered to have postoperative thrombosis. This study can benefit surgeons when dealing with patients who have risk factors for preoperative thrombosis (age ≥ 75 years old, female, D-dimer ≥ 0.5 mg/l, and revision THA), requiring more cautious surgical planning and standardized perioperative anticoagulation. Despite this, there were also some limitations. For starters, since this was a retrospective analysis and all relevant data was gathered for a single cohort, the study's incidence cannot accurately represent patients with common THA. Another point is that the number of DVT diagnoses, which was just 38 patients, was quite low. The numbers prevented the identification of several potentially significant concerns. A wider range of measures may be required for additional analysis because some crucial data, such as the preoperative hip score and hematological parameters, may be missing during data collection. Lastly, in this study, most positive DVT cases were located in the muscular veins, only 4 DVT cases were found in the proximal and calf-deep veins. In some previous studies, although the intermuscular vein does not belong to the main veins of the calf, studies have also shown that the intermuscular vein plays an important role in the formation of thrombosis and further evolution into PE, so it is still of unique significance for clinical work. Thus, we included intermuscular vein thrombosis as the positive result in this study.

## Conclusion

In conclusion, the incidence of preoperative DVT in non-fracture patients was 3.1%, with 2.4% in primary THA and 9.0% in revision THA. The independent risk factors for preoperative DVT in patients waiting for THA were age ≥ 75 years old, female, D-dimer ≥ 0.5 mg/l, and revision THA. When evaluating the related risk factors associated with DVT formation preoperatively, it is important to take these into account before THA.

## Data Availability

All data used during the study are available from the corresponding author.

## Abbreviations

DVT: Deep vein thrombosis
THA: Total hip arthroplasty
VTE: Venous thromboembolism
PE: Pulmonary embolism
CFV: Common femoral vein
SFV: Superficial femoral vein
PV: Popliteal vein
SV: Soleal vein
PEV: Peroneal vein
PTV: Posterior tibial vein
ATV: Anterior tibial vein
MV: Muscular vein
BMI: Body mass index
PT: Prothrombin time
INR: International Normalized Ratio
APTT: Activated partial thromboplastin time
TT: Thrombin time
Fbg: Fibrinogen
TG: Triglyceride
TC: Total cholesterol
HDL: High density lipoprotein cholesterol
LDL-C: Low density lipoprotein cholesterol
ApoA: Apolipoprotein A
ApoB: Apolipoprotein B
TKA: Total knee arthroplasty
